# Possible criteria for inpatient psychiatric admissions: which patients are transferred from emergency services to inpatient psychiatric treatment?

**DOI:** 10.1186/1472-6963-6-150

**Published:** 2006-11-22

**Authors:** Marc Ziegenbein, Christoph Anreis, Bernhard Brüggen, Martin Ohlmeier, Stefan Kropp

**Affiliations:** 1 Department of Social Psychiatry and Psychotherapy, Hannover Medical School, 30623 Hannover, Germany; 2 Department of Clinical Psychiatry and Psychotherapy, Hannover Medical School, 30623 Hannover, Germany; 3 Central Interdisciplinary Emergency Department, Hannover Medical School, 30623 Hannover, Germany; 4 Department of Psychiatry, Psychotherapy and Psychosomatics, Asklepios Fachklinikum Teupitz, 15755 Teupitz, Germany

## Abstract

**Background:**

Patients with psychiatric problems often seek help and assistance in hospital emergency departments. An important task of emergency room staff is to decide whether such patients need to be admitted or whether they can be treated on an outpatient basis.

**Methods:**

Psychiatric treatments given in the Central Interdisciplinary Emergency Department (CED) at the Medical University of Hannover (MHH) in 2002 were analysed.

**Results:**

Of a total of 2632 patients seeking psychiatric help, 51.4% were admitted for inpatient treatment. Patients with dementia syndromes were admitted more frequently than patients with other psychiatric diseases. Suicidality was often the reason for admission. Accompanied patients were less likely to be hospitalised, unless a care-order was in force. Restraining measures and acute medication also had an impact on the rate of admissions.

**Conclusion:**

The results may help psychiatrists in the emergency department to make a more effective decision regarding inpatient admission in the interest of the individual patient.

## Background

There are many paths that lead to inpatient psychiatric treatment: referral by a specialist, patients attending an emergency department, referral by social psychiatric services, patients brought in by the fire brigade and police, as well as transfers from somatic departments. For many patients with psychiatric problems, the first port of call is the emergency department of a hospital, which provides psychiatric assistance around the clock and usually also has a psychiatric department. For the attending doctors and nursing staff, work in an emergency department can present a special challenge, depending on the volume of patients, the severity of the cases, and other tasks that have to be performed at the same time. A major task confronting the staff in a hospital emergency department is the decision whether a patient needs to be admitted for inpatient treatment, or whether outpatient treatment is possible and sufficient. This important decision is made primarily on the basis of clinical assessment and the diagnosis rendered. However, other, possibly unknown or obscure criteria may also have an important influence on the rate of admissions. Neither in Europe, nor the USA are there any guidelines or recommendations concerning the indication for inpatient psychiatric admission [1].

The objective of this study is to provide an insight into existing influencing factors in a highly frequented emergency department in a German city. On the basis of these results, it may be possible to create better preconditions for improving the structure of care services for patients requiring inpatient psychiatric treatment.

## Methods

The basis for this retrospective investigation is formed by the emergency psychiatric treatments in the Central Emergency Department (CED) at the Medical University of Hannover (MHH) in 2002. The MHH is part of the statutory health-care system and, with its two psychiatric departments (Department of Social Psychiatry and Psychotherapy and Department of Clinical Psychiatry and Psychotherapy), serves two densely populated urban sectors of the state capital Hannover, with a population of around 142,000. The MHH has 104 psychiatric beds and 20 places in a day clinic, with individual specialist wards for addictive diseases, gerontopsychiatry, sociotherapy and psychotherapy. The primary distribution of the patients in the CED to the individual specialist disciplines is done by the nursing staff employed there. All patients who presented at the CED in 2002 and were examined by psychiatrists attending the CED were included in the study in anonymous form. This also took into account psychiatric consultancy provided for other specialist disciplines. The data collected originate from the documentation of the CED, the preliminary medical reports and the consultancy sheets. For all patients who were given a psychiatric examination in the CED and were thus included in the study, these data were entered in a data mask of the statistics program SPSS 14.0 designed for this purpose and analysed. Apart from descriptive frequency counts and calculations of means, depending on the test situation, either the non-parametric Mann-Whitney U-test or the Kruskal-Wallis H-test were used for calculating significance. Logistic regression (method = forward stepwise) was used to analyse the strength of the influence of the individual variables on the decision regarding admission.

## Results

### General data

A total of 34058 patients were treated in the CED at the MHH in 2002, whereby 2632 patient contacts were allotted to psychiatry (2069 patients primarily and 563 patients secondarily as consultancy for other specialist disciplines). Of these, 1839 cases were fully evaluable. By comparison, other disciplines had the following patient numbers: surgery: 9392 patients, internal medicine: 6478 patients, and neurology: 3313 patients.

The psychiatric patients comprised a total of 1359 men (51.6%) and 1273 women (48.4%). The average age of the total population was 43.5 years (+/- 16 years), the youngest patient was 15, the oldest 96 years. 2133 patients (81%) originated from sectors belonging to the city of Hannover, 205 (7.8%) came from the Hannover region, and 175 (6.6%) were resident outside the Hannover region. The address was not known for 119 of the patients (4.5%).

### Inpatient admission

945 patients, equivalent to 51.4% of the patients who presented in the CED (whose records were evaluable in this respect), were admitted to the psychiatric department of the MHH or to the psychiatric department of another hospital, directly after treatment in the CED. 106 patients (5.8%) were admitted to a somatic ward of the MHH or of another hospital.

### Outpatients

**Figure 1:**
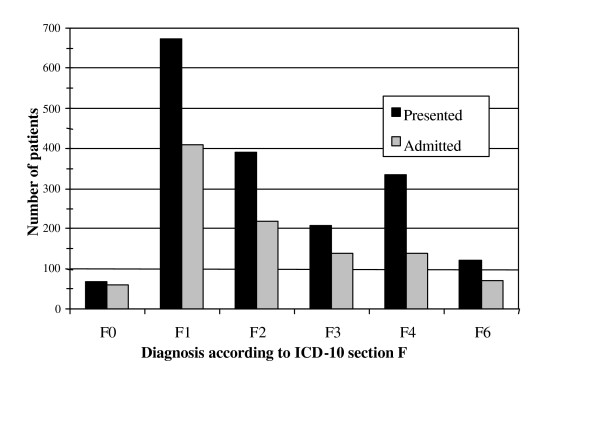
Number of patients presenting in the CED (black) and number of patients admitted from each diagnostic group (grey).

Of the remaining 788 patients (42.8%) who were not admitted, 674 were discharged after treatment in the CED, 50 patients (2.7%) left the MHH against medical advice, 57 patients (3.1%) left the CED without or before completion of treatment. In 7 cases (0.4%), patients had to be ejected from the CED by the police or security staff. The percentage distribution to the individual diagnostic groups is shown in Table 1. The diagnoses rendered in the CED and the patients admitted from each diagnostic group can be seen in Figure 1.

### Statistical results

The diagnosis rendered had a major influence on the decision in favour of inpatient treatment. Patients with a demential disease were admitted highly significantly more often than average (p < 0.001). A trend towards inpatient treatment was determined in patients with an F3 diagnosis, but this was not significant.

Acute suicidality (222 patients) or a condition after attempted suicide (106 patients) frequently led to inpatient admission (p = 0.000). Drug intoxication (45.8%), cutting (26.2%) and jumping from hights (5.6%) were the most common methods of suicide attempts. With regard to the diagnostic group and suicidality, a further correlation was found here: Suicidality was present highly significantly less often in patients with F0 and F2 diagnoses (p < 0.001). Patients with the diagnostic groups F3 (p < 0.001), F4 (p < 0.05) and F6 (p < 0.001) showed a significantly or highly significantly higher than average incidence of suicidality.

Whether patients came to the CED on their own or accompanied by others also had an influence on admission to inpatient treatment: Patients who were accompanied by relatives, the police, ambulance services, or the like, were admitted significantly less often than patients who presented at the CED on their own (p = 0.000). Independently of this, patients under a legal care order (guardianship) were admitted more often than patients who were not under legal supervision (p = 0.001). Patients who brought a referral letter from a medical practice with them were admitted in 79.5% of the cases, whereas only 53.5% of the patients without a referral were admitted (p = 0.000).

In addition, the influence of a committal order (Involuntary hospitalization according to the german law on assistance and precautionary measures in cases of mental illnesses) on the admission rate was investigated, whereby there was a significant difference to the patients without a committal order (p = 0.000). Analysis of the influence of restraining measures on the admission rate also produced a clear result (p = 0.000). Of 29 patients who were restrained by force either before or during treatment in the CED, 28 were admitted. A further factor to be mentioned is the acute medication administered in the CED: Patients who received medication were admitted highly significantly more often than patients who did not receive medication (p = 0.000).

The rate of admissions on the individual days of the week did not show any significant differences (p = 0.252), although there was a difference between the admission rate of 58.6% on workdays and 52.8% at weekends (p = 0.019). There was also a difference with regard to inpatient treatment according to the psychiatric treatment sector to which the patients belonged. 98.1% of the patients from the two sectors of the MHH were admitted here if they required inpatient treatment. But as many as 45.4% of patients from other sectors were also admitted to the MHH if they so requested. The most important variables that influenced the decision on admission were then further analysed by logistic regression. The variables that have higher odds ratios for inpatient admission can be found in Table 2, the predictors for outpatient follow-up treatment in Table 3. The predictive probability for the situation "admission" or "outpatient treatment" is 80.7% and the model explained 57.2 percent of the variance.

## Discussion

The collected data provide clear evidence for answering the question regarding possible criteria for an indication for inpatient psychiatric admission. Various influences on the indication were found, which are mostly consistent with clinical experience, although to our knowledge no current figures are known or have been published for the individual hospitals in Germany. As one of the classical psychiatric emergency indicators, suicidality is a major factor influencing the decision on admission. Recent guidelines (NICE and APA) and different studies point out that among the factors found to predict suicide, a previous suicide attempt is one of the strongest [2,3]. It is well kown that most suicide cases and most of those who attend an emergency department following an act of self-harm meet criteria for one or more psychiatric diagnoses at the time they are assesed [4,5]. Concerning suicidal behaviour our results are in line with other studies [6]. Hirschfeld [7] showed that the descision to hospitalize patients at imminent risk for suicide requires careful assesment of risk factors. The emergency department provides the most services for people who self-harm and proper assesment, monitoring, and treatment of patients with imminent risk for suicide save lives. Another major factor influencing the decision on admission is a committal order issued by the authorities, whereby the committal itself must be seen as the expression of a severe mental illness. As a sign of acute disease, drug treatment initiated in the emergency department can also be regarded as an indicator of the necessity for inpatient treatment. Similarly, restraining measures taken appear to be an expression of the need for inpatient treatment. As a general rule, they only have to be applied in the case of outward aggression, but they are simply signs of the acuteness of a disease. Apart from the acute disease, welfare considerations and social aspects appear to play a role in the admission situation both in demential diseases and in general. We have no other way of explaining the fact that unaccompanied patients are admitted much more often than those who come to the emergency department accompanied by other people. The helplessness of the patients concerned certainly may be a variable that determines the indication for admission. However, this point cannot be analysed in more depth on the basis of the data at our disposal. The high admission rate of patients with a referral from practising physicians is also understandable, since inpatient treatment has already been considered as a last resort in an outpatient setting and has been suggested to the patient. That not all patients with a referral were ultimately admitted may be due to the fact that the referrals were not always issued by psychiatric specialists and that the indication for inpatient admission could be avoided in some patients in the view of the emergency physicians by taking other measures.

The high admission rate of patients with dementia might be related to other medical conditions which also need inpatient treatment and assessment. These patients are usually more severely impaired patients with more severe cognitive disorders, poor nutritional status and were most dependent for basic activities of daily living. The number of diagnostic procedures and consultations also had an impact on the rate of admissions. Often consultations by other faculties and diagnostic procedures are needed in psychiatric patients with somatic comorbideties. Women were significantly more likely to have had a psychiatric hospitalization than men. This circumstance could be related to the differential use of mental health services by men and women.

Whereas data from other research groups, e.g. in the USA, have been used to show that the indication for admission may be determined not so much on the basis of clinical or demographic data, but by external circumstances and the wish for social control, our results show that it is above all severely ill, suicidal or helpless patients who are hospitalised [8]. In marked contrast to other studies, it is unaccompanied patients who tend to be admitted for inpatient treatment in our population [8]. Reasons for the high admission rate of unaccompanied patients might be that these patients often have little or no support at home. The very important information from other sources is not available, including family members and friends. Such individuals may be able to provide information about the patients current mental state, activities, and psychosicial crises and may also have observed behaviour or been privy to communications from the patient that suggest suicidal ideation, plans or intention. Stravynski and Boyer [9] found a significant correlation between experiencing suicidal ideation or attempting suicide and living alone and having no friends. Unusually high compared to other investigations is the number of patients who were admitted for inpatient treatment here, at over 50% [10-12]. Although the emergency department is open to all patients, inpatient admissions are distributed to the responsible hospitals according to the psychiatric sectors, but over 40% of the patients from foreign sectors with the established indication were still able to be admitted to the MHH at their own request. On the basis of this initial review of the situation, attempts will now be made to improve existing processes in the emergency department always in the direction of more patient-oriented treatment.

## Conclusion

Suicidality, drug treatment administered in the emergency department, restraining measures applied, committal ordered under state laws, the diagnosis of dementia, the number of consultations, female gender, referral to hospital by a physician, or the patient presenting at the emergency department unaccompanied are the main factors that favour the indication for inpatient admission in our study. With increasingly limited time and high personal demands on the individual, a knowledge of these factors may provide doctors working in emergency departments with important pointers to help them more quickly and efficiently select the appropriate form of psychiatric treatment for the individual patient. In order to gain further insights in this field, further studies should be conducted, which should also include such aspects as the influence of the length of clinical experience of the duty physician on the number of inpatient admissions.

## Competing interests

The author(s) declare that they have no competing interests.

## Authors' contributions

SK and MZ conceived and designed the evaluation and helped to draft the manuscript. CA participated in designing the evaluation and performed parts of the statistical analysis. MO re-evaluated the clinical data and revised the manuscript. CA and BB collected the clinical data, interpreted them and revised the manuscript. All authors read and approved the final manuscript.

## Pre-publication history

The pre-publication history for this paper can be accessed here:



## Figures and Tables

**Table 1:** Relative incidence of the diagnostic groups in relation to the total population. Missing diagnoses 1.7%

Diagnostic group	Percentage of the total group
F0 (Organic, including symptomatic, mental disorders)	3.8
F1 (Mental and behavioural disorders due to psychoactive substance use)	36.5
F2 (Schizophrenia, schizotypal and delusional disorders)	21.3
F3 (Mood [affective] disorders)	11.3
F4 (Neurotic, stress-related and somatoform disorders)	18.1
F5 (Behavioural syndromes associated with physiological disturbances and physical factors)	0.5
F6 (Disorders of adult personality and behaviour)	6.6
F7 (Mental retardation)	0.1
F9 (Behavioural and emotional disorders with onset usually occurring in childhood and adolescence)	0.1

**Table 2:** Significant variables in the logistic regression predicting a psychiatric admission on the basis of the analysed total population.

Variable	Beta	S.E.	Wald	df	p-value	OR	Cl (95%)
Suicidality	2.470	0.213	134.66	1	0.000	11.81	7.78–17.93
Diagnosis of dementia	0.672	0.316	4.51	1	0.034	1.95	1.05–3.63
Female sex	0.252	0.116	4.72	1	0.030	1.28	1.02–1.61
Two diagnostic measures	0.513	0.239	4.61	1	0.032	1.67	1.04–2.66
One consultation	0.476	0.156	9.35	1	0.02	1.61	1.18–2.185
Two consultations	1.281	0.300	18.22	1	0.000	3.60	2.00–6.485
Care order in force	0.528	0.224	5.54	1	0.019	1.69	1.09–2.633
Referral by a doctor	0.809	0.221	13.37	1	0.000	2.24	1.45–3.462
Medication in the emergency department	1.094	0.217	25.43	1	0.000	2.98	1.95–4.566
Patient reffered by a consultant	1.273	0.158	65.17	1	0.000	3.57	2.62–4.863

-2 Log likelihood 1904.547, Cox & Snell R Square 0.387, Nagelkerke R Square 0.520

**Table 3:** Significant variables in the logistic regression predicting outpatient follow-up treatment on the basis of the analysed total population.

Variable	Beta	S.E.	Wald	df	p-value	OR	Cl (95%)
Diagnostic group F4	- 0.953	0.151	39.85	1	0.000	2.59	1.93–3.48
Stay of over 3 h	- 0.660	0.165	16.075	1	0.000	1.93	1.40–2.67
No diagnostics	- 2.216	0.281	62.31	1	0.000	9.17	5.29–15.90
One diagnostic measure	- 2.328	0.212	120.27	1	0.000	10.25	6.76–15.55

-2 Log likelihood 1904.547, Cox & Snell R Square 0.387, Nagelkerke R Square 0.520
